# Medical Opinion and Movement

**Published:** 1911-04-15

**Authors:** 


### Medical Opinion and Movement.

### Vaccination and Eczema.

In view of the general opinion that it is undesirable to vaccinate in cases of eczema and other skin affections, it is interesting to record the experiences of Dr. Chiperskaia, assistant to Dr. Koulnev, professor of dermatology in the Military Academy of Medicine at St. Petersburg. In the course of an epidemic of smallpox 76 patients in the hospital, most of them children, were vaccinated. There were 18 cases of eczema (in 4 cases the affection involved the face, in 4 the hands, and in 10 the body), 15 cases of lupus of the face or the extremities, 20 cases of herpes tonsurans, and others of psoriasis, prurigo, etc. In all these patients the vaccine pustules followed a perfectly normal course, and there was not a single case of general vaccinia. On the other hand, the vaccination had no injurious effect upon the eczema or any of the other skin affections. Even in the case of some of the infants, who were affected with eczema over a large area of the body-surface, there was no aggravation of the condition. These facts would tend to show that the complications of eczema which have been attributed to vaccination result from other causes, probably from failure of asepsis, allowing the introduction of pyogenic organisms into the system.

### Fractured Lower Jaw.

Dr. L. H. Landry describes in the *New Orleans Medical Journal* a very simple and at the same time efficient method of treating complicated fractures of the lower jaw, in which intrabuccal prosthetics are contra-indicated. It was originally suggested by Dr. Matas. It consists in making five or six turns of a thin Esmarch bandage round the head and chin, bringing the crowns of the teeth of the lower jaw in contact with those of the upper, and in this manner splinting the lower jaw against the upper, and at the same time favouring the reduction and absorption of the hæmatoma and œdema. Dr. Landry reports details of a case treated in this manner. The lower jaw was fractured on both sides, most of the teeth were loosened, and there was much swelling. As the immobilising bandage naturally tends to promote salivary stagnation and putrefaction, special attention must be given to the cleansing of the mouth. This was carried out by means of an irrigating can attached to the head of the bed and provided with a female douche-nozzle, which the patient was instructed to use himself every hour while awake, allowing the solution to go in as far back as possible and flow outward. In two or three days the swelling had subsided enough to allow the patient to take soft food. In another two days all the acute symptoms were so far reduced that he was allowed to walk about. The bandage was removed and re-adjusted whenever necessary to wash the skin. After seventeen days the patient left the hospital with only a slight deformity and a good functioning jaw.

### Diabetes and Cane-sugar.

At a meeting of the Académie des Sciences, Dr. J. Le Goff drew attention to the great increase in mortality from diabetes in Paris and in the Departement de la Seine. According to the *Annuaire statistique de la ville de Paris*, the number of deaths from diabetes in 1880 was 128, or 0.644 per 10,000 inhabitants; in 1885 this had increased to 261, or 1.165 per 10,000. This increase has continued so that in 1900 the mortality amounted to 427, and in 1909 to 525, or nearly 2 deaths per 10,000 inhabitants; thus in thirty years the number of deaths from diabetes has quadrupled. It cannot be supposed that the mortality of the disease has increased to this extent without a considerable increase in the number of cases, so that one may conclude from this great increase in mortality in thirty years that there has also been a marked increase in the number of patients suffering from the disease. It may also be suggested that the disease was not so well recognised thirty years ago, but the author points out that the methods of analysing the urine for the detection of sugar have not varied much during the last fifty years, and certainly before 1880 the method of detecting sugar in the urine by Fehling's solution was already well known and practised. The author suggests that the causes for this increase in the disease are to be found in the sedentary life of so many of the inhabitants and in the great increase in the consumption of cane-sugar which has taken place during the whole of the nineteenth century. According to the author, it is precisely in those countries where there is the greatest consumption of cane-sugar that one finds the largest number of diabetics.

### Lumbar Puncture.

In a contribution to *Le Journal de Médecine de Paris*, Dr. Le Filliatre discusses the important question of the seat of election for lumbar puncture. It is generally held that the seat of election for lumbar puncture is the fourth intra-vertebral lumbar space. According to some, however, it is of no consequence which intra-vertebral space is chosen. Tuffier considers that either the third or fourth intra-vertebral lumbar space may be chosen. He points out that the arachnoido-dural space between the second lumbar and second sacral vertebræ is free from the spinal cord and only contains the nerves of the cauda equina, which are mobile and easily escape the needle. Even if they are pricked no serious result follows. Now according to the anatomical researches of Dr. Filliatre these nerves of the cauda equina do not lie free in the space, but are fixed and massed together on the postero-lateral wall of the lumbo-sacral canal, the arachnoid forming a separate special sheath to each of the nerves. There is, therefore, a space in the lumbar region which is surrounded by the arachnoid and the nerves of the cauda equina and the filum terminale, and is only filled with cerebro-spinal fluid. The author proposes to call this the "lac arachnoido-lumbar." It commences in the adult generally at the level of the first intervertebral lumbar space and ends at the junction of the second and third sacral vertebræ. The transverse diameter is almost constant as far as the first sacral vertebra and is about 32 millimetres. The antero-posterior diameter is 11 millimetres at the third lumbar vertebra and enlarges to 17 or 18 millimetres at the fifth. According to the author, puncture at the fourth space can easily prick the fifth lumbar nerve-roots, causing a sharp pain down the leg. But at the fifth space the fifth pair of lumbar nerves are no longer present and the other branches of the sacral plexus are sufficiently posterior to escape the needle. The fifth lumbar or sacro-lumbar space has the further advantage of giving a wider field for operation: the transverse diameter between the apophyses is 38 mm. against 28 mm. in the fourth space, and the vertical measure of the sacro-lumbar space is also considerably greater than that of the two intra-vertebral spaces above. The advantages of the lumbo-sacral space are, therefore, that it is larger, that the arachnoid space here is deeper antero-posteriorly, and there is no danger of injury to the nerves of the cauda equina.

### Test Tubes v. Infant's Stomach.

In the *Archives of Pediatrics*, Cowie and Lyon narrate some very interesting experiments which they have carried out to ascertain how far the digestive processes as studied in test tubes correspond to those that actually occur in the stomachs of living babies. They find that all the digestive juices are secreted from the first day of life. Free hydrochloric acid, however, rarely occurs in the stomachs of infants during the active part of the day, though it may occur sometimes in the fasting stomach. The evacuation of the normal infant's stomach depends to a large extent upon the degree of acidity of the stomach contents; but excessive acidity delays evacuation. A total "acidity value" of 8 to 30 offers the normal stimulus for pyloric relaxation. Basic calcium casein does not inhibit the formation of rennet curds in the infant's stomach. It delays the formation of curd, the saturation of the proteid in the acid, and the acid pyloric reflex; and thus delays the evacuation of the stomach contents. The action of rennet on basic calcium casein in the test-tube is not comparable with that which takes place in the stomach, as the conditions are not the same. Sodium citrate, however, inhibits the action of rennet both in the stomach and in the test-tube. The curds formed in the stomach—like that formed *in vitro* with dilute acid and citrated milk—are casein hydrochloride curds. But the curds normally present in the infant's stomach are paracasein hydrochloride.

### A New Method of Politzerisation.

Politzer, in a recent communication to *Die Therapie der Gegenwart*, proposes to introduce a modification into the procedure which he invented for inflating the ear drum through the Eustachian tube. As usually practised, the bag is made use of while the patient is swallowing. Politzer now proposes to use it while the patient is making very deep and prolonged inspiration. After introducing the nozzle into one of the nares of the patient, and then closing the nostrils with the thumb and first finger of the left hand, the physician tells the patient to pucker up his lips as if about to whistle, and then through the rounded opening so made to take in a deep and prolonged breath of air. At the same time the physician presses the bag hard, so that the air passes into the Eustachian tubes. If the patient is unable to purse his lips as desired, a piece of rubber tubing may be placed between them, so that he may draw air through it. In the course of such an inspiration the naso-pharyngeal isthmus closes, and the Eustachian tube opens. The author states that this new method succeeds in cases where the old one has failed to produce a clear passage to the drum, and that it clears away the naso-pharyngeal mucus more effectively from the openings of the tubes than does any other procedure. Moreover, he states that it can be used with safety in cases of catarrh of the middle ear, and in the later stages of acute otitis media when the symptoms of reaction and pain have ceased, whereas catheterisation and violent blasts of air during the process of swallowing —as in the old method of using the bag—are contraindicated.

### Cocaine Poisoning.

The *Veterinary Record* publishes the summary of an article which appeared in the *Berliner Tier Woch.*, from the pen of Pelz of Leipsig, relative to the use of Esmarch's bandage as a precaution against cocaine poisoning. This observer, working on rabbits, confirms Kohlhardt's results as regards the efficiency of the bandage, but explains its action differently. The latter observer infers that the tissues which are detached from the circulation exercise a special influence upon the cocaine to lessen its activity. Pelz, however, believes that the action of the ligature is explained by altered conditions of absorption, by the formation with the tissues of cocaine compounds, and by the affinity of the erythrocytes for cocaine. He summarises his results as follows: (1) Care should always be exercised in the use of cocaine, for even small doses exert a toxic action. The drug causes venous congestion in the abdominal cavity, and anæmia of all other vascular areas. It is possible that the alterations of the red blood discs within the vessels from the action of the drug may be harmful. (2) The simultaneous application of cocaine to various parts of the body should be avoided. (3) Esmarch's bandage prevents general intoxication by cocaine more certainly than the simultaneous use of adrenalin, especially if the cocaine is injected into the deeper-lying tissues, and care be taken to adjust the bandage properly. (4) In cases where the bandage is inapplicable, either general narcosis or a less dangerous local anæsthetic, such as alypin, should be used. This conclusion, again, is especially applicable in the case of the deeper-lying structures.

